# Wheelbarrow tire explosion causing trauma to the forearm and hand: a case report

**DOI:** 10.1186/1752-1947-3-129

**Published:** 2009-11-16

**Authors:** Mark Lenz, Ralf Schmidt, Thomas Muckley, Torsten Donicke, Reinhard Friedel, Gunther O Hofmann

**Affiliations:** 1 Department of Trauma, Hand and Reconstructive Surgery, Friedrich-Schiller-University Jena, Germany

## Abstract

**Introduction:**

Tire explosion injuries are rare, but they may result in a severe injury pattern. Case reports and statistics from injuries caused by exploded truck tires during servicing are established, but trauma from exploded small tires seems to be unknown.

**Case presentation:**

A 47-year-old german man inflated a wheelbarrow tire. The tire exploded during inflation and caused an open, multiple forearm and hand injury.

**Conclusion:**

Even small tires can cause severe injury patterns in the case of an explosion. High inflating pressures and low safety distances are the main factors responsible for this occurrence. Broad safety information and suitable filling devices are indispensable for preventing these occurrences.

## Introduction

Tires should be considered as compressed air tanks. However, substantial safety regulations set up for the operation of pressure tanks are not applied to tires. Tire explosion injuries are rare, but may result in a severe injury pattern. We report an open multiple forearm and hand injury from an exploded wheelbarrow tire during inflation.

## Case presentation

A 47-year-old german man inflated a wheelbarrow tire with a filling device at the gas station. The tire exploded. As a result, his left forearm and left hand were fractured. In particular, a second degree open complete diaphyseal forearm fracture AO type A3 [1], a distal radius fracture AO type B1, a fracture of the fourth metacarpal and of the second middle phalanx basis were diagnosed. The blood supply to and sensibility of his left hand were not affected before or after surgery. In particular, no acute entrapment neuropathy, which would have required immediate decompression, was found. A preventive cutting of his transverse carpal ligament was not performed due to the presence of closed soft tissues in this region.

Skin hematoma and superficial wounds were located on his head and neck. A computed tomography scan of the patient's cranium and cervical spine revealed no other lesions.

**Figure 1:**
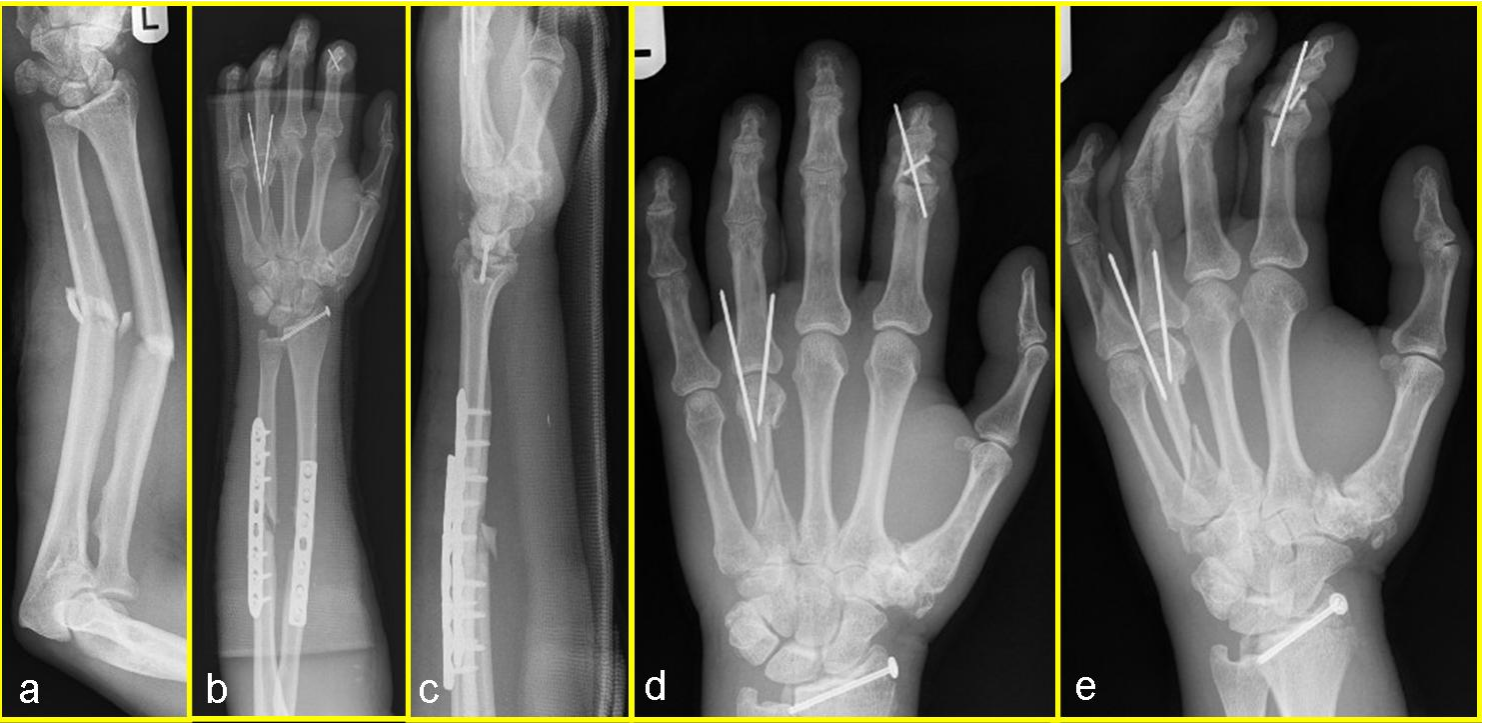
**(A) Preoperative image of the fracture of the forearm shaft and distal radius**. (B) Image of the fracture of the forearm shaft and distal radius after fixed-plate osteosynthesis. (C) Image of the fracture after the screw osteosynthesis. (D) Image of percutanous Kirschner wire osteosynthesis of the 4th metacarpal. (D) Image of percutaneous Kirschner wire osteosynthesis of the 2nd middle phalanx basis.

Cefuroxime was administered to the patient as an antibiotic prophylaxis. After the immediate debridement of the soft-tissue injury, the forearm fracture was stabilized with a 3.5 mm fixed-angle plate. The distal radius fracture was fixed with screw osteosynthesis (Figure 1) and primary wound closure of the patient's forearm was achieved. Apart from superficial wounds, the integument of the hand was closed with concomitant soft-tissue swelling which led us to fix the metacarpal and phalanx fracture with Kirschner wires. The postoperative X-ray (Figure 1) showed a stable osteosynthesis of the forearm and distal radius allowing immediate mobilisation. The metacarpal and the middle phalanx fracture were fixed sufficiently with Kirschner wires. The postoperative soft-tissue swelling decreased under physiotherapy. Intensive in-patient and out-patient physiotherapy and ergotherapy were performed. The postoperative follow-up after one year revealed a complete bone union, a wrist motion of extension and flexion of 50/0/30°, and a radial deviation and ulnar deviation of 25/0/15°. The patient's forearm rotation was reduced to a pronation and supination of 90/0/50°. Compared with the contralateral side, the grip strength on the patient's injured hand diminished to 10 kp.

## Discussion

Explosion injuries occur occasionally and are mainly due to firecrackers, home-made explosive devices or industrial and domestic explosives. Injuries from tire explosions are rare but may result in severe trauma. Tire explosions usually involve truck tires, especially multi-piece rim wheels. An evaluation conducted by a German employers' liability insurance association (Berufsgenossenschaften) [2] recorded 89 accidents caused by exploding tires between 1989 and 1999, including nine lethal accidents. However, these figures involved only the accidents registered at the insurance association, and the actual number of accidents caused by inflating car tires in leisure time would be higher.

Occupational safety devices like a protection cage with an automatic inflating gadget will help minimize the risk of injury from tire inflation. A safety distance of 2.5 metres from the inflating tire is recommended. If the wheel is not fixed, its components including the wheel rim could act as missiles. Big, exploding tires can produce blast waves. These risks are mainly unknown to the general public, so safety instructions are often ignored. Besides the underestimation of such a potential hazard in tire inflation, other risks involve damaged tires and wheel rims, overpressure for tire setting, and short inflating hoses.

The literature mostly consists of case reports and retrospective analyses of exploded truck tires [3-7]. The average pressure is 8 to 9 bars for truck tires, 2.5 to 3 bars for car tires and 2 to 2.5 bars for wheelbarrows and pushcarts. For bikes the pressure rises, depending on the cross-sectional tire width, from 2 bars (cross bike, mountain bike) up to 9 bars (racing bike). Accidents due to exploding small tires or tires with low pressure are not found in the literature.

Exploding truck tires mainly cause severe facial and eye injuries [6] and intracranial lesions or limb trauma [4,6]. Only a few articles deal with upper limb trauma due to exploding truck tires [3,5,7]. Luxations of the interphalangeal joints, and phalanx and metacarpal fractures are described [5]. As in our patient, most fractures are open fractures with concomitant soft-tissue damage. Unfortunately, these case reports are limited to the extent of the trauma and its operative treatment and do not comment on follow-up procedures.

The extent of the trauma is determined by the explosion, the transmission medium, and the distance to the explosion focus [8]. The damage is caused by the explosion pressure and hurtling particles. The thermal and chemical damage, usually an essential component of explosion injuries, can be neglected. In our patient, treatment was performed according to the principles of explosion injuries [9]. A good blood supply to the tissue, an eradication of debridement of the necrotic structures, and primary antibiotic prophylaxis are essential for a good wound healing and for the avoidance of secondary complications like infections. Often, an extensive debridement is necessary when primary wound closure is not feasible. In our patient, primary wound closure was achieved. This, however, should not be done in situations with extreme contamination and extensive tissue damage. Nevertheless, during the first operation vital tissue should be protected as much as possible to achieve maximum function with minimum secondary operations, as postulated by Kleinert [10].

Tissue damage is the most important factor in treating explosion injuries. Osteosynthesis has to be adapted when treating tissue damage. Like the patients of Matloub *et al.*[5], our patient received osteosynthesis for the forearm (plate osteosynthesis) and hand (k-wires). Although plate osteosynthesis of the hand bones [5] is a possible alternative, we chose the k-wire fixation because of our patient's soft-tissue swelling and closed integument.

Hand fractures indicate that extensive forces affected the hand. Especially in this injury pattern, therefore, possible further tissue damage must be taken into account. Each tissue structure reacts in a different way to an explosion trauma. The injury mechanism caused by a sudden pressure variation mainly affects liquid-filled cavities like the vessels, muscle fascias and tendon sheaths [11]. Elastic structures like vessels and nerves appear to be macroscopically intact although they could be damaged. Nerve injuries have a good prognosis [12]. Our patient's postoperative blood supply was not disturbed or interrupted. Periodical examination of a patient's blood supply is crucial in the first postoperative days, because the blast wave or hurtling parts can induce long intimal tears that may require a secondary adventitectomy or venous interposition grafts.

**Figure 2:**
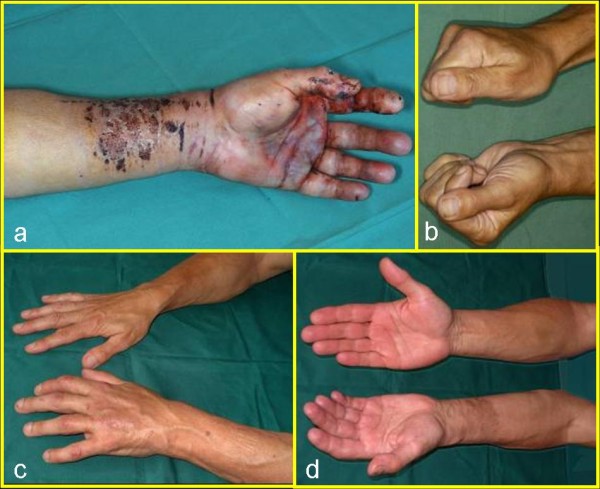
**(A) Image of the volar skin hematoma and superficial wounds of the forearm indicating the local force effect**. (B) Image of the postoperative result with nearly complete fist closure. (C) Image of forearm rotation with full pronation. (D) Image of forearm rotation with slightly reduced supination.

Explosion injuries may result in multiple flexor tendon ruptures [13] that arise from the effect of sudden force on the flexed hand, as when provoked by an exploding firecracker held in the closed hand. Interestingly, we did not find a flexor tendon rupture in our patient. The volar-located superficial wounds suggest that the force was transmitted proximal to the hand, which caused consecutive complete forearm fracture without rupture of the tendons (Figure 2).

The massive tumescence of the injured forearm and hand observed in the first postoperative days was attributed to tissue damage. Even at this early stage, physiotherapy and ergotherapy proved indispensable to avoid the development of a limited range of motion. Stable osteosynthesis is mandatory for early mobilization. With intensive and consistent aftercare we were able to achieve a good outcome (Figure 2).

Comprehensive security advices showing the risks of tire inflation, safe inflating gadgets suitable for general use, and the performance of maintenance work by a trained tire vulcanizer may help minimize resultant severe injuries. Another option is the use of airless polyurethane foam tires, which are already available for wheelbarrows and other low-velocity applications.

## Conclusion

Dangerous explosions can happen even when servicing small tires. The main causes are high inflating pressures and low safety distances. Prevention can only be achieved through broad safety information and the use of suitable filling devices. Injuries of the fingers and the metacarpus have a direct influence on occupational rehabilitation, most notably in manual workers.

## Consent

Written informed consent was obtained from the patient for publication of this case report and any accompanying images. A copy of the written consent is available for review by the Editor-in-Chief of this journal.

## Competing interests

The authors declare that they have no competing interests.

## Authors' contributions

LM drafted the manuscript, assisted in surgery, and performed follow-up examinations on our patient. SR and DT performed the surgery. MT, FR and HG participated in the design of the study, performed the coordination, and revised the manuscript. All authors read and approved the final manuscript.
